# Radiosurgery for ventricular tachycardia: preclinical and clinical evidence and study design for a German multi-center multi-platform feasibility trial (RAVENTA)

**DOI:** 10.1007/s00392-020-01650-9

**Published:** 2020-04-18

**Authors:** Oliver Blanck, Daniel Buergy, Maren Vens, Lina Eidinger, Adrian Zaman, David Krug, Boris Rudic, Judit Boda-Heggemann, Frank A. Giordano, Leif-Hendrik Boldt, Felix Mehrhof, Volker Budach, Achim Schweikard, Denise Olbrich, Inke R. König, Frank-Andre Siebert, Reinhard Vonthein, Jürgen Dunst, Hendrik Bonnemeier

**Affiliations:** 1 Klinik für Strahlentherapie, Universitätsklinikum Schleswig-Holstein, Campus Kiel, Arnold-Heller-Straße 3, Haus 50, 24105 Kiel, Germany; 2 Klinik für Strahlentherapie und Radioonkologie, Universitätsmedizin Mannheim, Universität Heidelberg, Medizinische Fakultät Mannheim, Mannheim, Germany; 3 Universität zu Lübeck, Zentrum für Klinische Studien, Lübeck, Germany; 4 Institut für Medizinische Biometrie und Statistik, Universitätsklinikum Schleswig-Holstein, Campus Lübeck, Lübeck, Germany; 5 Klinik für Innere Medizin III, Abteilung für Elektrophysiologie und Rhythmologie, Universitätsklinikum Schleswig-Holstein, Campus Kiel, Kiel, Germany; 6 Medizinische Klinik I, Abteilung für Elektrophysiologie und Rhythmologie, Universitätsmedizin Mannheim, Universität Heidelberg, Medizinische Fakultät Mannheim, Mannheim, Germany; 7 Medizinische Klinik mit Schwerpunkt Kardiologie (CVK), Abteilung für Elektrophysiologie und Rhythmologie, Charité Universitätsmedizin Berlin, Berlin, Germany; 8 Klinik für Radioonkologie und Strahlentherapie, Charité Universitätsmedizin Berlin, Berlin, Germany; 9 Institut für Robotik und Kognitive Systeme, Universität zu Lübeck, Lübeck, Germany

**Keywords:** Radioablation, Radiosurgery, Stereotactic body radiotherapy, SBRT, Ventricular tachycardia, Cardiac arrhythmia, Clinical feasibility trial, Multi-center, Multi-platform

## Abstract

**Background:**

Single-session high-dose stereotactic radiotherapy (radiosurgery) is a new treatment option for otherwise untreatable patients suffering from refractory ventricular tachycardia (VT). In the initial single-center case studies and feasibility trials, cardiac radiosurgery has led to significant reductions of VT burden with limited toxicities. However, the full safety profile remains largely unknown.

**Methods/design:**

In this multi-center, multi-platform clinical feasibility trial which we plan is to assess the initial safety profile of radiosurgery for ventricular tachycardia (RAVENTA). High-precision image-guided single-session radiosurgery with 25 Gy will be delivered to the VT substrate determined by high-definition endocardial electrophysiological mapping. The primary endpoint is safety in terms of successful dose delivery without severe treatment-related side effects in the first 30 days after radiosurgery. Secondary endpoints are the assessment of VT burden, reduction of implantable cardioverter defibrillator (ICD) interventions [shock, anti-tachycardia pacing (ATP)], mid-term side effects and quality-of-life (QoL) in the first year after radiosurgery. The planned sample size is 20 patients with the goal of demonstrating safety and feasibility of cardiac radiosurgery in ≥ 70% of the patients. Quality assurance is provided by initial contouring and planning benchmark studies, joint multi-center treatment decisions, sequential patient safety evaluations, interim analyses, independent monitoring, and a dedicated data and safety monitoring board.

**Discussion:**

RAVENTA will be the first study to provide the initial robust multi-center multi-platform prospective data on the therapeutic value of cardiac radiosurgery for ventricular tachycardia.

**Trial registration number:**

NCT03867747 (clinicaltrials.gov). Registered March 8, 2019. The study was initiated on November 18th, 2019, and is currently recruiting patients.

**Graphic abstract:**

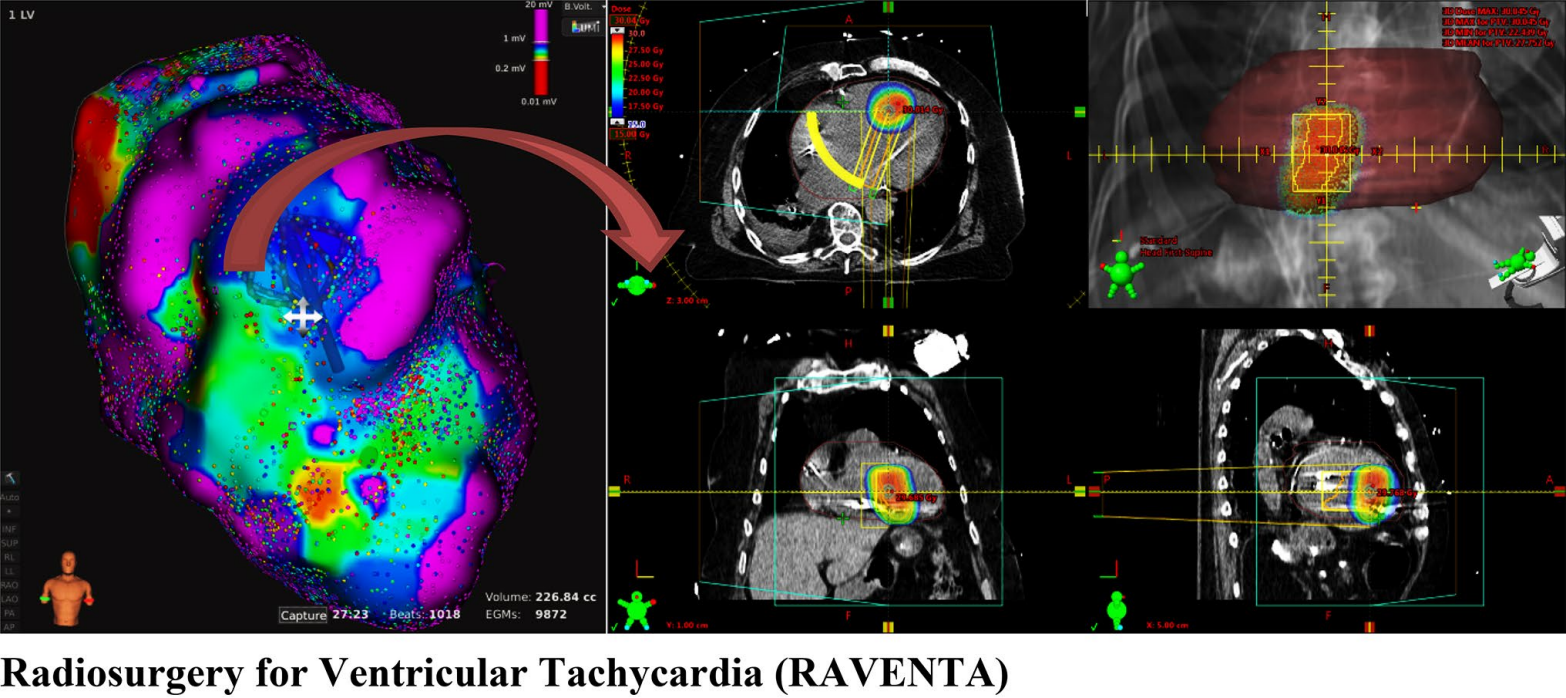

## Background

### Ventricular tachycardia

An abnormal electrical focus or circuit in the ventricular myocardium corresponding to scar regions after myocardial infarction can be the origin of ventricular tachycardia (VT) [1, 2]. The management of VT requires a rapid risk assessment of sudden death, of the extent of possible underlying heart disease as well as an evaluation of possible therapeutic options. The initial treatment consists of antiarrhythmic drugs and placement of an implantable cardioverter defibrillator (ICD). If drugs in adequate dosing fail to limit the VT episodes, catheter ablation of the arrhythmogenic substrate is the standard treatment option for VT arising around myocardial scars [1–3]. For selected patients, catheter ablation improves the composite outcome consisting of death at any time or VT storm or appropriate ICD shock 30 days after treatment [4]. However, up to half of the patients experience VT recurrence within 6 months even after initially successful catheter ablation [5]. Furthermore, the short-term morbidity and mortality rate of ablation procedures can be as high as 5% in the first 31 days after treatment and even higher for repeat procedures [6]. On the other hand, side effects in patients undergoing dose-escalated anti-arrhythmic drug treatment were even higher than in patients who were treated with catheter ablation instead [5, 6].

Given unsatisfying success rates even after repeated catheter ablation, high incidences of toxicities of antiarrhythmic drugs and an increasing number of patients not eligible for catheter ablation (due to co-morbidities or location of the VT substrate), there is an urgent need for alternative, non-invasive treatment approaches for the treatment and prevention of refractory VT. Stereotactic body radiotherapy (SBRT) in a single session to the heart, also called radioablation (RA) or cardiac radiosurgery (CRS), may overcome the limitations of endocardial or epicardial thermal ablation energy deposition and hence offer a treatment alternative for otherwise untreatable patients [7].

### Radiosurgery and stereotactic body radiotherapy

Already in the 1950s, frame-based intracranial stereotactic radiosurgery was evaluated as a high-precision high-dose radiotherapy procedure with steep dose gradients towards critical organs for the treatment of brain tumors. Since then, intracranial stereotactic radiosurgery (SRS, 1 treatment session) and intracranial fractionated stereotactic radiotherapy (FSRT, 2–12 treatment sessions) have become an integral part in the treatment of single and multiple brain metastases, also in combination with immune- and targeted-therapies [8], benign tumors (e.g., meningioma, acoustic neuromas, or pituitary adenomas) [9], vascular malformations and functional disorders (e.g., trigeminal neuralgia) [10], among others. With recent advances in image guidance and motion management for organ and target movements (e.g., due to respiration), extracranial stereotactic body radiotherapy (SBRT, 1–12 treatment sessions) has also become a standard treatment option for many indications including primary lung, liver, pancreas, kidney, and prostate cancer [11–13], as well as oligometastases in lung, liver, bone, and abdominal localization [14–16]. Due to rapid technical development of this method in the past 2 decades, SBRT now allows a highly precise dose deposition in any target in the body with steep dose gradients to surrounding healthy organs.

The idea to use SBRT to ablate the substrate of cardiac arrhythmias originated from Thomas J. Fogarty (Stanford, USA) and resulted in the first patent to treat atrial cardiac arrhythmia non-invasively in 2003 [17]. After several preclinical studies were conducted [18], the first-in-human patient treatments for VT and atrial fibrillation (AF) were performed in 2012 and 2015, respectively [19, 20]. For VT, the main goal of SBRT is to ablate the arrhythmogenic substrate with an adequate dose and minimal safety margins using steep dose gradients to avoid cardiac radiation toxicity [21]. As RA/CRS is a novel therapeutic approach, several issues regarding target volume definition, target volume transfer between cardiac and radiation oncology treatment (planning) systems, cardiac and respiratory motion assessment and management techniques, optimal target dose, and critical organ maximum and volume dose limitations and follow-up have to be further optimized in a dedicated interdisciplinary RA/CRS team and the treatment has to be assessed in terms of safety and efficacy in close controlled and monitored clinical trials.

## Methods/design

### Clinical trial and literature review

Prior to trial design, we searched for ongoing and completed clinical trials using the European Union Clinical Trial Register (EU CTR, www.clinicaltrialsregister.eu) and the United States National Library of Medicine Clinical Trial Register (USNLM CTR, www.clinicaltrials.gov) and performed a systematic literature search for preclinical and clinical data using the USNLM PubMed/Medline database (PMD, www.ncbi.nlm.nih.gov) complemented by own pioneering technical, preclinical, and clinical experience in the past 12 years.

For the EU CTR, we used the following search terms: (1) “Ventricular Tachycardia” AND “SBRT”, (2) “Ventricular Tachycardia” AND “Stereotactic Body Radiation Therapy”, (3) “Ventricular Tachycardia” AND “Radiosurgery”, (4) “VT” AND “SBRT”, (5) “VT” AND “Stereotactic Body Radiation Therapy” and (6) “VT” AND “Radiosurgery”. For the USNLM CTR, we searched the conditions “Ventricular Tachycardia” OR “Arrhythmia” with other terms “SBRT” OR “Stereotactic Body Radiation Therapy” OR “Radiosurgery” OR “Noninvasive Ablation” without any other restrictions. For PMD, we used the search terms (1) “Ventricular Tachycardia” OR “VT” OR “Atrial Fibrillation” OR “AF” in definitive combination with “SBRT” OR “Stereotactic Body Radiation Therapy” OR “Radiosurgery” OR “MR Linac” OR “Particle Therapy” OR “Proton” OR “Heavy Ion” OR “Noninvasive Ablation” OR “Radioablation” with the restriction of publication after 1st January 2007. The search was initially performed in December 2018 and updated in August 2019, the latter being the basis of the following results.

Ten clinical feasibility trials are currently registered, seven of them in the USNLM CLR, the presented RAVENTA trial (NCT03867747) included. One clinical trial with 20 patients from St. Louis (USA) has already been published (NCT02919618 [22, 23]), while another longer term ongoing study from Austin (USA) with 10 patients has not yet published results (NCT02661048). On the other hand, two similar trials (NCT03601832 and NCT03819504) with different mapping techniques for target definition (invasive endocardial electro-anatomic mapping with 50 patients and non-invasive external body electrophysiological mapping with 10 patients) have recently started accruing patients in Ostrava (Czech Republic). Further studies who recently started or are in preparation are from Milan (Italy) with 15 patients (NCT04066517), Isehara (Japan) with 3 patients (jRCTs032190041), Calgary (Canada) with 20 patients (NCT04065802), and Amsterdam (The Netherlands) with 6 patients (NL7510). For atrial fibrillation, only one trial has been conducted in Tokyo (Japan) with three patients (UMIN000031322), though the results have not been published in a peer-reviewed journal yet.

The PMD search revealed 263 unique publications. For further assessment, all manuscripts not published in English and not related to cardiac radiosurgery by means of abstract screening were removed. Finally, we added initial technical, pre-clinical, and clinical manuscripts not covered by the search based on our own experience in cardiac radiosurgery in the past 12 years. Evidence was finally extracted from the remaining 42 manuscripts, which include reports on two clinical trials [22, 23], two clinical case series [24, 25], eight case reports [19, 20, 26–31], eight preclinical studies [18, 32–39], fourteen technical studies [40–53], and eight reviews or opinions [7, 54–60].

### Preclinical/animal studies for cardiac radiosurgery

The first preclinical studies for RA/CRS were the studies from CyberHeart Inc. [18, 32], the company that owned several patents for performing cardiac radiosurgery until 2018 ([17], among others). The first study published by Sharma et al. was mainly exploratory; the authors reported on irradiation of different locations in the hearts of 16 Hanford–Sinclair mini swine with various doses [18]. The studies showed that cardiac radiosurgery can produce cavotricuspid isthmus block, AV nodal block, and decreased voltage at the pulmonary vein-left atrial junction with single fraction treatment. A second publication by Maguire et al. revealed more details in terms of electrophysiology and pathology correlations of focused PA/CRS to the pulmonary veins in the same animal model to treat AF [32]. Although the authors claimed that both 25 Gy and 35 Gy in a single fraction can lead to electrical block and transmural fibrosis, only data for the 35 Gy treatment were shown. A third publication by Zei et al. [37] presented the same data as in Maguire et al. [32] with the additional data of RA/CRS targeted to the pulmonary veins in 17 canines. The authors found treatment effects for doses between 15 and 35 Gy during electrophysiology studies and gross pathological analysis [37]. Unfortunately, baseline and detailed histopathological data of those new treatments were not provided, while the dosimetry for some animals was presented elsewhere [46]. On the other hand, a more technically oriented publication by Gardner et al. from the same animal studies showed that RA/CRS with carefully selected motion management can be delivered precisely to the heart (within 5–6% dose accuracy) using several implanted dosimeters [33].

Nevertheless, a lot of questions remained from those early studies which two groups from Germany tried to address. Our group used photon irradiation to find the needed threshold dose for RA/CRS and treated 13 Göttingen minipigs with single fraction doses of 15–40 Gy in 2.5 Gy dose steps in a randomly assigned, investigator-blinded animal study with baseline measurements [34, 36]. The studies showed that for transmural fibrosis and electrical block at the pulmonary vein antrum of the healthy minipigs, a minimum dose of 32.5 Gy is needed. On the other hand, a lethal bronchial fistula occurred at a dose of 40 Gy in one minipig. The other group pursued the use of heavy ions instead of photons for RA/CRS [35, 38, 39]. In principle, particles have the major advantage over photons that the maximum dose intensity delivered per beam can be varied in depth at the so-called Bragg Peak, while for photons, the maximum dose is always close to the body surface (approximately 1.5 cm for 6 MeV photons). The authors conducted various animal experiments and reported histopathological findings in great detail, yet they also found that much higher doses are needed (> 25 Gy) to generate therapeutic effects [38, 39].

### Technical studies for cardiac radiosurgery

Technical studies for RA/CRS must be divided into treatment planning and treatment delivery studies, while, of course, one is closely connected to the other and vice versa. Treating a small volume within a (mostly rapidly) beating heart that also displaces due to respiration is challenging. There are already various studies published for catheter ablation which investigated the cardiac and respiratory motion of cardiac substructures to obtain a better registration between electrophysiological measurements and pre-procedural imaging [61]. Additionally, for RA/CRS, two studies investigated the motion of the left atrium and the pulmonary veins, and found highly variable respiratory and cardiac motion ranges and also differential motion between left, right, anterior, and posterior pulmonary veins [45, 47]. However, with most modern radiotherapy equipment, the major source of motion (namely originating from respiration) can be compensated to a high degree of accuracy [62], while cardiac contraction motion seems to have a limited impact on the dose distribution [53].

Nevertheless, all forms of active motion compensation require continuous motion detection and, due to latency of the radiotherapy systems, also motion prediction [40]. Since real-time motion detection is desired, several studies investigated the use of MRI for cardiac target localization and multileaf collimator tracking [41, 48, 49, 51]; some patients have already been treated on such machines (reports pending and personal communications). In the technical MR studies, the authors found that locating and tracking targets in the heart are feasible with an accuracy of < 5 mm. This seems to be in line with the robotic radiosurgery system used in the early animal studies; however, this system required implanted fiducial markers or radiopaque landmarks in or near the target [33], while MRI does not. Other methods of motion compensation include ultrasound tracking [63], gating [64], or both [65]; however, they have not been systematically tested for RA/CRS yet.

The spatial and dosimetric accuracy of different systems used for RA/CRS mainly drives treatment planning as technical uncertainty margins are needed to compensate for residual errors in treatment delivery. Additionally, the minimum effective dose needs to be determined. There are three studies which demonstrated that 25 Gy delivered to the pulmonary antrum to treat AF can only be considered safe if (a) 25 Gy is indeed the minimum treatment effective dose, (b) the uncertainty margins can be kept at 3 mm or lower, and (c) the esophagus is centrally located at the left atrium posterior wall and does not move during treatment [41, 44, 45]. Due to these constraints, the potential treatment of AF would be technically difficult, even if different beam energies or heavy ions (which are unavailable in most centers) are used [42]. On the other hand, RA/CRS for VT seems to be much easier to plan dosimetrically due to (a) larger distance between the target and radiosensitive critical structures (25 Gy applicable in most cases), (b) limited cardiac and differential motion (smaller uncertainty margins possible), (c) fiducial marker already near or in the target area (i.e., the ICD leads), and (d) pre-existing cellular damage at baseline in target cells which may decrease the minimum dose required to achieve clinically relevant effects (possibly < 25 Gy) [43, 50, 52].

Furthermore, the question arises which platform is the best suited system technically and dosimetrically, in this case for RA/CRS. The answer is quite simple nowadays as all dedicated SBRT systems can be considered equal dosimetrically based on multi-platform studies [66, 67], even though there seem to be some contradictory results for VT RA/CRS [52], and, technically, as long as active motion compensation is used [68]. For passive motion compensation techniques, the risk of additional dose to the heart and surrounding structures must be considered which, however, highly depends on the actual motion ranges in the individual patient under consideration of the motion management strategy applied (e.g., when using abdominal compression, the additional respiratory motion margins may be small [24]).

### Clinical data for cardiac radiosurgery

In 2012, the first RA/CRS patient treatment of VT was performed in Stanford (USA) (reported in 2015) [19], followed by a second patient treated in 2014 in Ostrava (Czech Republic) [26]. Both patients received a single fraction radiation dose of 25 Gy to the VT substrate, as defined by electroanatomical voltage mapping and showed only limited-to-no toxicity with a significant reduction in VT burden and ICD shock frequency after treatment. The clinical treatment effects with 25 Gy were remarkable, given the results from the animal models, in which 25 Gy showed only limited-to-no effects on electrical block and transmural fibrosis [32–39]. It might be hypothesized that modulation of cardiac conduction rather than induction of transmural fibrosis explains the treatment effect seen after RA/CRS with 25 Gy. However, the role of preexisting fibrosis and the local microenvironment in arrhythmogenic substrates is currently unknown. Since then, five more case reports have been published for VT RA/CRS [27–31] and more are likely under review. In those case reports, RA/CRS as rescue procedure for electrical storm from VT [27] or fibrillation [30] or as a treatment option for recurrent VT with high-resolution mapping [31] or secondary to a cardiac fibroma [28] or lipoma [29] have been reported. On the other hand, only one case report for RA/CRS for AF has been reported until today, and even though the results seem to indicate scarring based on late enhancement MRI, the actual AF was still persisting [20].

Apart from the case reports, two case series [24, 25] and two reports of one clinical trial (ENCORE-VT) [22, 23] have been reported. Most impressively, Cuculich et al. (St. Louis, USA) reported the use of RA/CRS for five patients who had recurrent VT after catheter ablation or who were ineligible for a catheter ablation [24]. In the 3 months prior to treatment, the patients had a mean number of 1,315 VT episodes (range 5–4312). Following a 6-week blanking period after RA/CRS, the number of VT episodes was reduced by 99.9% and no major complications during or immediately after the treatment were reported. In one patient with atrial fibrillation, risk factors for thromboembolic events and contraindications to anticoagulants, a fatal stroke occurred during follow-up [24]. Histologic assessment of the myocardium did not show any major signs of fibrosis or necrosis in the treatment area, an observation which is in line with the previous animal study results with 25 Gy. The subsequent clinical phase I/II trial from the St. Louis group confirmed the initial results [22]. Here, a VT burden reduction of more than 75% was found in 89% of the patients. Furthermore, a reduction of dual anti-arrhythmic medication from 59 to 12% was possible with a significant improvement in quality of life. The overall survival at 1 year was 72%, a result which would be expected in such a population. On the other hand, 10.5% of the patients developed a severe adverse event (grade 3) in the first 90 days after treatment [22] and overall survival was reduced with larger target volumes as demonstrated in a more detailed dosimetric analysis [23]. Finally, longer term retrospective follow-up of 10 patients treated with RA/CRS in Ostrava (Czech Republic) was recently reported [25]. Here, VT burden was significantly reduced by 87.5% and only minimal side effects were noted (three cases of mild nausea and one case of gradual progression of mitral regurgitation). In the Czech study, target volumes were considerably smaller as compared to the ENCORE-VT trial and 80% of the patients had recurrent VT as assessed by the authors after the blanking period of 6 weeks.

### Study design and patient selection

**Fig. 1:**
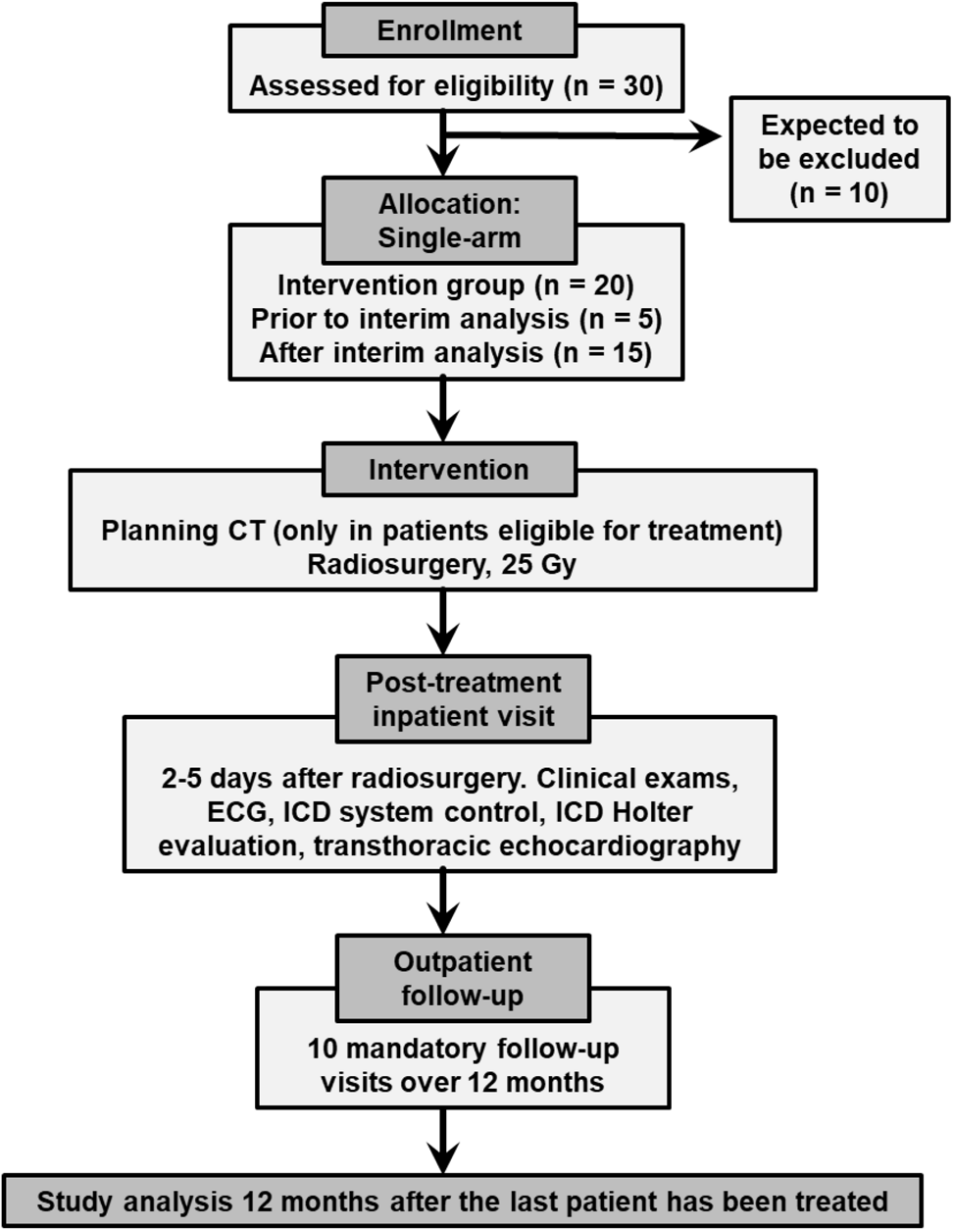
Consolidated standards of reporting trials (CONSORT) diagram for the RAdiosurgery for VENtricular Tachycardia (RAVENTA) trial

Eligible for the RAVENTA trial are only patients that are refractory to dose-escalated anti-arrhythmia drug treatment and where catheter ablation (endo- and/or epicardial) has either already been performed or is deemed to be unsuccessful (e.g., due to the location of the VT substrate) or associated with high risks (e.g., due to clinical co-morbidities or the patient). Due to the novelty of the method and the very limited clinical experience, we developed a feasibility study which will be conducted as a multi-center study with a central quality control (consolidated standards of reporting trials diagram presented in Fig. 1). The toxicity profile is yet not well known. After discussions in our group and with the regulatory authorities, we came to the conclusion that safety is the only appropriate endpoint for this initial feasibility study, even though many further questions for RA/CRS need to be answered. Foremost, the main question besides safety is which patients will benefit the most from this new treatment modality. It is likely that frail patients who are in need for a local treatment will derive a relevant benefit from this new treatment option, because all other treatment options have failed. On the other hand, patients with a very limited life expectancy have to be excluded, to allow for a minimum follow-up period of 6 months or more in the majority of patients.

The key inclusion criteria for the RAVENTA trial are:

- Patients older than 18 years with structural heart disease,
- life expectancy > 6 months and,
- implantable cardioverter defibrillator (ICD) with,
- symptomatic monomorphic ventricular tachycardia that requires ICD intervention (e.g., shock or anti-tachycardia stimulation) and which is,
- refractory to antiarrhythmic medication therapy in maximal dose and,
- (a) recurring with at least three episodes within three months prior to inclusion or,
- (b) inducible by ICD via non-invasive programmed stimulation (NIPS) or during electrophysiology measurement, or,
- (c) both (a) and (b).

The key exclusion criteria for the RAVENTA trial are:

- Lack of evidence of a myocardial scar triggering the ventricular tachycardia, or,
- ICD electrode malfunction, e.g., impedance, sensing amplitude, pacing threshold out of range, or,
- prior radiation therapy to the thoracic region, or,
- pregnancy or breastfeeding, or,
- any other contraindication to radiosurgery (e.g., precise target volume definition not possible due to image artifacts created from a left-ventricular assist device).

### Study treatment

For this first feasibility study, the treatment will be performed as an inpatient procedure or as outpatient produce with immediate inpatient submission after treatment. Cardiac radiosurgery will be prescribed, recorded, and reported on the basis of the International Commission on Radiation Units and Measurements (ICRU) report 91 for stereotactic treatments with small photon beams [69, 70]. The essential data for CRT are the time-resolved non-contrast enhanced primary planning computed tomography (PCT), the contrast enhanced multi-phase ECG-gated cardiac CT (CCT), and electrophysiology mapping (EPM) data of the right or left ventricle. The term “time-resolved” in this context means that the PCT acquisition will be adapted according to the SBRT motion management technique [e.g., PCT performed at computer-controlled inspiration for deep inspiration breath hold (DIBH) gating or robotic real-time tracking or as 4D CT for passive motion compensation strategies]. The CCT will be registered with the primary PCT and capture the full cardiac motion (e.g., left ventricle position in end systole and end diastole).

**Fig. 2:**
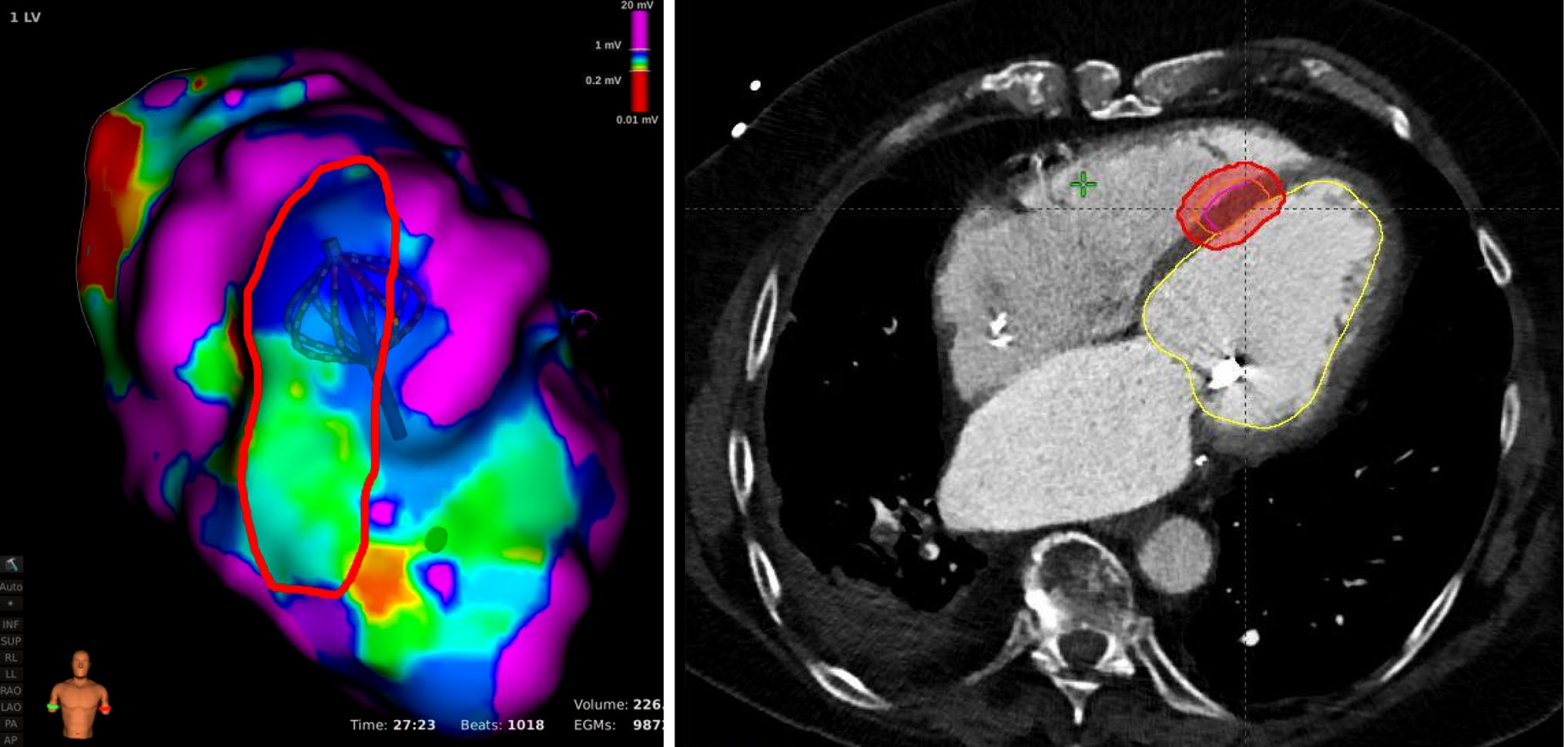
High-resolution electroanatomical voltage mapping (left) showing a re-entry circuit of approx. 3.6 × 1.5 cm in the cardiac septum (red circle) and corresponding axial plane in the radiation treatment planning system (right) showing the target lesion in the septum (orange circle) and the planning target volume which included a 5 mm uncertainty margin (red circle)

The target region will primarily be defined from the EPM procedure(s) and include the VT substrate as determined by the treating electrophysiologist (example presented in Fig. 2). This shall be done by marking the VT substrate region as 2D surface in the EPM software. The target region, which basically consists of endocardial measurement points that are registered to the CCT, will then be transferred to or re-created on the primary PCT. This could either be done via dedicated registration and visualization software (if available) or done by manually re-creating the target surface region from the EPM (with registered CCT) on the co-registered PCT using 3D contouring on the segmented left ventricle in the radiation treatment planning systems (RTPS) according to the current clinical standards [19, 20, 22–31]. For the latter, an in-house system available to participating centers will be used to jointly display both the target region from the EPM and the re-created surface from the RTPS. Finally, the target surface will be expanded transmurally into the ventricle wall in the RTPS to create the final target volume (TV). Information from a 12-lead ECG and other functional imaging modalities (i.e., cardiac MRI, PET/CT, and myocardial scintigraphy) will be used to additionally guide this process, if available. The planning target volume (PTV) will then comprise the TV including a respiratory and cardiac motion range and technical treatment margin according to the planning imaging data and best-practice guidelines of the RTPS (see Fig. 2).

Radiosurgery will be performed as described previously [22–24] as single fraction treatment with a dose of 25 Gy prescribed to the 95% PTV-encompassing isodose line (PTV D95% ≥ 25 Gy). There is currently no evidence from any human or preclinical studies that may point at the possible benefits of dose reduction [34] or increase [22–25], though future clinical trials may explore the possibility to reduce the full target dose or the dose to parts of the target for selected patients. Contrary to the previous studies and supported by preclinical studies [34], we allow higher maximum doses within the TV. The near-maximum dose is limited to 32.5 Gy (PTV D2% ≤ 32.5 Gy), with dose peaks exceeding 30 Gy being desirable, though exclusively centered in the TV (TV *D*_max_ ≥ 30 Gy and PTV − TV *D*_max_ ≤ 30 Gy). Dose-limiting constraints to nearby critical structures are well described in the literature [19, 22–26, 32, 34, 43–45, 50, 71], yet dose limits from radiosurgery to compartments in the heart and heart substructures such as valves or papillary muscles are largely unknown and have only been recently reported though not yet correlated to toxicity [23]. Dose limits for the RAVENTA trial are presented in Table 1. One minor, but no major violation to the dose limits is allowed for the RAVENTA trial. This may result in the exclusion of patients from the trial after treatment planning (e.g., if the PTV is close or overlapping with coronary arteries or the stomach).

**Table 1:** Organs at risk dose recommendations and dose limitations

Organs at risk	Dose recommendations/dose limitations
Aorta	Dose limitations: *D*_max_ ≤ 20.0 Gy Minor protocol deviation: 20 Gy < *D*_max_ ≤ 25 Gy Major protocol violation: *D*_max_ > 25 Gy
Left coronary arteries	Dose limitations: *D*_max_ ≤ 14.0 Gy Minor protocol deviation: 14 Gy < *D*_max_ ≤ 20 Gy Major protocol violation: *D*_max_ > 20 Gy
Superior vena cava	Dose recommendations: *D*_50%_ ≤ 0.6 Gy
Left atrium	Dose recommendations: *D*_max_ ≤ 4.4 Gy
Whole heart minus PTV	Dose recommendations: *D*_50%_ ≤ 5 Gy
Esophagus	Dose limitations: *D*_max_ ≤ 14.5 Gy and *V*_9Gy_ ≤ 1 ccm Minor protocol deviation: *D*_max_ ≤ 19 Gy, *D*_1ccm_ ≤ 14.5 Gy and *V*_9 Gy_ ≤ 4 ccm Major protocol violation: *D*_max_ > 19 Gy || *D*_1ccm_ > 14.5 Gy || *V*_9 Gy_ > 4 ccm
Trachea	Dose limitations: *D*_max_ ≤ 15 Gy and *V*_10 Gy_ ≤ 1 ccm Minor protocol deviation: *D*_max_ ≤ 20 Gy, *D*_1 ccm_ ≤ 15 Gy and *V*_10 Gy_ ≤ 4 ccm Major protocol violation: *D*_max_ > 20 Gy || *D*_1 ccm_ > 15 Gy || *V*_9 Gy_ > 4 ccm
Bronchial tree	Dose limitations: *D*_max_ ≤ 15 Gy and *V*_10Gy_ ≤ 1 ccm Minor protocol deviation: *D*_max_ ≤ 20 Gy, *D*_1 ccm_ ≤ 15 Gy and *V*_10 Gy_ ≤ 4 ccm Major protocol violation: *D*_max_ > 20 Gy || *D*_1 ccm_ > 15 Gy || *V*_9 Gy_ > 4 ccm
Spinal canal	Dose limitations: *D*_max_ ≤ 7 Gy and *V*_6 Gy_ ≤ 0.1 ccm Minor protocol deviation: *D*_max_ ≤ 8 Gy, *V*_6 Gy_ ≤ 1 ccm Major protocol violation: *D*_max_ > 8 Gy || *V*_6 Gy_ > 1 ccm
Skin	Dose limitations: *D*_max_ ≤ 14.4 Gy and *V*_10 Gy_ ≤ 10 ccm Minor protocol deviation: *D*_max_ ≤ 16 Gy, *V*_14.4 Gy_ ≤ 10 ccm Major protocol violation: *D*_max_ > 16 Gy || *V*_14.4 Gy_ > 10 ccm
Whole lungs	Dose limitations: *V*_100% _− *V*_7Gy_ ≥ 1500 ccm (*V*_7 Gy_ remaining volume > 1500 ccm) and *D*_5%_ ≤ 20 Gy and *D*_50%_ ≤ 3.5 Gy Minor protocol deviation: *V*_100% _− *V*_7Gy_ ≥ 1000 ccm (*V*_7 Gy_ remaining volume > 1000 ccm), *D*_6.5%_ ≤ 20 Gy and *D*_50%_ ≤ 5 Gy Major protocol violation: *V*_100% _− *V*_7 Gy_ < 1000 ccm (V_7 Gy_ remaining volume < 1000 ccm), *D*_6.5%_ > 20 Gy and *D*_50%_ > 5 Gy
ICD (major electronics)	Dose limitations: *D*_max_ ≤ 0.5 Gy and blocked from primary beam irradiation Minor protocol deviation: 0.5 Gy < *D*_max_ ≤ 1.0 Gy Major protocol violation: *D*_max_ > 1.0 Gy

All SBRT techniques/platforms available at the meantime are well capable to generate highly conformal treatment plans anywhere in the body [42, 66, 67], though we are restricting the use of higher energies (maximum 6 mega electronvolt) for cardiac radiosurgery due to the presence of an ICD in these patients [72, 73]. Plan optimization will be performed using the best-practice guidelines for each system according to the ALARA (as low as reasonably achievable) principle for critical structures inside and outside of the heart. Due to radiation biology considerations, a treatment (beam-on) time of less than 20 min is desirable (e.g., using volumetrically modulated treatment techniques and flattening filter-free beams) to minimize the influence of cell-repair mechanisms under prolonged irradiation [74]. The actual treatment will be performed under close cardiological monitoring (continuous ECG monitoring and standby cardiac emergency team during treatment) according to the guidelines of the DGK (German Society of Cardiology) and DEGRO (German Society of Radiation Oncology) [72]. Patient repositioning and target localization will be performed by means of integrated image guidance ideally using a time-resolved or triggered volumetric cone-beam CT (CBCT) according to the motion management strategy. Alternatively, stereoscopic X-ray images may be used for image guidance insofar as landmarks near the target (e.g., ICD leads) can be precisely located and used for motion compensation. Directly after treatment the ICD system will be evaluated for functionality and will be reprogrammed, if necessary. Follow-up of the patients consisting of clinical evaluation, ICD checks and Holter monitor readout, transthoracic echocardiography and electrocardiography will be performed in the first 5 days daily after treatment, with hospital discharge ideally in this time, and 15 and 31 days, 6 and 8 weeks, and 4, 5, 6, 9, and 12 months after cardiac radiosurgery. Additionally, a thoracic CT will be performed at 3 months after treatment to assess possible asymptomatic radiation pneumonitis and pericarditis.

### Study objectives

The main objective of the RAVENTA trial is to evaluate safety of RA/CRS for VT. The primary endpoint is to assess the 30-day post-intervention safety defined as presence of both, complete radiosurgery delivery of the planned dose to the intended target area, and no possibly treatment-related serious adverse events (grade ≥ 3) in the first 30 days after treatment. This primary endpoint was chosen to ensure the early study termination which should be any major concern in the immediate time after radiosurgery arise. Based on the previous clinical data [19, 20, 22–31] for this endpoint, we assume that RA/CRS is feasible and safe in at least 70% (study stopping rule) and ideally in ≥ 95% of the treated patients.

Secondary study endpoints that will be evaluated within the first year of follow-up are:

- reductions in ventricular tachycardia episodes and ICD interventions,
- reductions in antiarrhythmic medication due to treatment effects,
- occurrence of possibly treatment-related adverse events,
- changes in patient-reported quality of life, and
- overall survival.

Episodes of VT and ICD interventions will be assessed through Holter monitor readout. A blanking period of 6 weeks after treatment for the radiation effects to set in will be set forth and antiarrhythmic medication will be subsequently reduced if indicated by the reductions of VT episodes and ICD interventions. Adverse events will be assessed according to the Common Terminology Criteria for Adverse Events (CTCAE, version 5.0) and classified based on causality to RA/CRS with the categories: (a) no causal relationship, (b) unlikely causal relationship, (c) possible causal relationship, and (d) likely or definitive causal relationship. It will be of specific importance to distinguish between progression of the underlying heart disease and treatment-related adverse events. In unclear cases that are not ascribable to any of both, an adverse event will be assumed. These classifications are similar to the first larger clinical trial which already published detailed results [22]. Quality of life will be recorded through the EQ-5D-5L questionnaire and overall survival will be captured as any patient contact. Due to the limited data, patients will also be followed after study completion of each patient and of the study to obtain further long-term safety data.

### Statistical planning

Based on Simon and Fleming's two-stage designs, an interim analysis is performed after the first 5 included patients have been assessed for the primary endpoint. Accordingly, the inclusion of additional patients cannot be carried out until at least 30 days after the completion of treatment of the fifth patient. The sample size planning is based on 95% success for the primary endpoint (i.e., less than or equal to 5% possibly related serious adverse events within 30 days after delivery of the full planned dose), significance level of 5% and a power of 90%.

In a two-stage test procedure with multiple significance level of nominal 0.05 and a power of nominal 0.90 after observing 5 and possibly 20 cases, safety is tested. An insufficient safety of 70% (null hypothesis H0) should quickly and safely lead to the rejection of the hypothesis of a safety of 95% (H1), but with a probability of stopping for futility below 0.5. However, if the safety is 95%, then this should ultimately lead to the rejection of the null hypothesis of insufficient safety. If the safety is in between, the statement is confined to the confidence interval. Thus, the expected number of patients assuming H0 is 12.9, because with a probability of 0.47, the study will terminate after 5 patients, and if H1 is true, it is 19.7 patients. This design differs from the Simon two-stage design in that it does not minimize the expected number of patients, but does the interim analysis for the smallest possible number of cases. Calculations used R package gsDesign, function gsBinomialExact. Loss to follow-up is part of the primary endpoint.

The analysis strategy is the composite strategy of ICH E9 (R1) Addendum on Estimands, i.e., loss to follow-up, and intercurrent events like rescue LVAS will be counted as a lack of both, safety and feasibility. Toxicity is summarized in a graph as cumulative frequencies of the toxicity levels for each time point. Mortality is presented as a Kaplan–Meier curve. Effectiveness is described by measures of location and scatter (95% confidence intervals) of the distributions of the features and differences to baseline. In general, those will be confidence intervals derived from the score function for proportions and Hodges–Lehmann intervals for medians. The dimensions of quality of life are represented as trajectories; supplemented by a median trajectory, with missing values being imputed by the worst value in the same patient. For the quality-of-life index determined according to the published weights, averages and distributions are given for the individual times and for the areas under the curves. Adverse events are tabulated by organ system, intensity, and relatedness.

### Trial duration and study termination

The patient accrual is estimated to be completed within 2 years after start (estimated start in November 2019), with a follow-up period of 1 year after the inclusion of the last patient. The clinical trial should be concluded 3 years after first inclusion. Early termination of the trial will occur if the toxicity of the performed RA/CRS is determined to be unacceptable for any possibly related serious adverse events during the study (i.e., adverse events ≥ grade 3 in the first month after treatment in more than one of the first five or more than two out of ten patients). The study can be terminated early, if (a) the hypothesis of the study can be answered by new data, or (b) events observed later after treatment are more frequent than must be expected given published data, or (c) the recruiting target is not met during the recruitment phase.

### Ethical and legal considerations, quality harmonization, and patient safety

As RA/CRS is deemed an experimental therapy for VT, the RAVENTA trial needed authority approval which has been obtained from the German National Radiation Protection Authority (Bundesamt für Strahlenschutz, BfS, reference number Z5-22463/2-2018-054) and from the leading ethics committee of the University of Kiel (reference number 555-18), and subsequently by the ethics committees responsible for further participating centers. The clinical trial registration number at clinicaltrials.gov is NCT03867747. The study is monitored and audited by the center for clinical trials (ZKS Luebeck, Germany, protocol number ZKS-121-09). Sponsor of the study is the University Medical Center Schleswig Holstein (UKSH).

With respect to clinical trial harmonization for this experimental therapy, the evaluation criteria and examination protocols, as well as materials such as test sheets are uniform for all centers. Furthermore, the documentation for commissioning, dose calibration, and quality assurance of the respective radiosurgery treatment system are centrally reviewed in accordance with the national standards and laws. An independent absolute dose audit for each treatment system is mandatory every 2 years. A benchmark study consisting of target volume definition and treatment planning for exemplary VT RA/CRS cases is ongoing, though the initial results were already discussed. In brief, general consensus was reached between the centers; however, it was decided to perform a planning target volume and treatment plan joined group interdisciplinary review within the participating centers for all patients before treatment to quickly harmonize and improve as a whole group.

**Fig. 3:**
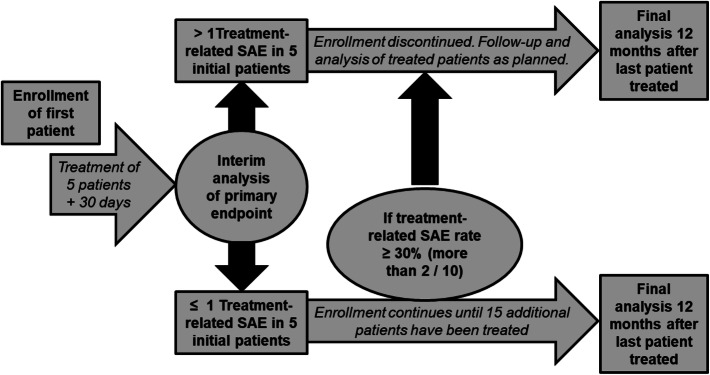
Flowchart for the RAdiosurgery for VENtricular TAchycardia (RAVENTA) trial

Concerning patient safety for this experimental therapy, multiple safety layers were implemented within the RAVENTA trial (flowchart presented in Fig. 3). For one, the study team decided jointly with the authorities, and that the first five patients in the study will be treated sequentially in each center based on primary endpoint assessment by an independent data and safety monitoring board (DSMB). In each center, further patients can only be scheduled for treatment if the last treated patient was assessed for the primary 30-day post-treatment endpoint by the DSMB. After five patients are treated in the trial, an interim analysis is performed as described above and further clinical trial continuation is determined jointly with the DSMB, the auditors, the study team, and the authorities. Besides frequent patient assessment during follow-up, additionally, a thoracic CT is performed 3 months post-treatment for each patient to assess possible asymptomatic pneumonitis and pericarditis. Finally, the study termination rules are set strictly as described above to maximize patient safety.

## Discussion

Due to the novelty and the very limited clinical experience of radioablation/cardiac radiosurgery (RA/CRS) for ventricular tachycardia (VT) [19, 20, 22–31], standardized clinical trials are urgently needed. However, a discussion on the proper primary endpoint of such trials is necessary. Currently, there are a growing number of case reports and small retrospective case series in the literature [19, 20, 24–31], but only one “phase I/II trial”, i.e., pilot-stage clinical investigation which reported results so far [22, 23]. The primary endpoint of this single-center trial (NCT02919618) was safety and the authors demonstrated that RA/CRS for VT is feasible. However, they did notice some possibly treatment-related, yet manageable, severe side effects (grade 3), with a limited number of patients and only short-term follow-up. Furthermore, there are four ongoing prospective clinical trials: two also focusing primarily on safety (NCT02661048, NCT04066517) and two trials from the same institution focusing on efficacy as a primary endpoint (NCT03601832 and NCT03819504). At the current stage of development, we decided that safety is the preferable primary endpoint until sufficient data are generated in a multi-center and multi-platform setting which is the main goal of the RAdiosurgery for VENtricular TAchycardia (RAVENTA) trial (NCT03867747). In this regard, we worked closely with local and federal authorities to minimize possible harm to patients with an independent data and safety monitoring board (DSMB) and accredited auditors, sequential treatment in each center, and an early interim analysis with overall strict study termination rules. However, it should be emphasized that safety issues occurring in less than 10% of patients may not be detected due to the small-sample size.

Apart from safety and feasibility, the RAVENTA trial will generate further insights regarding the effect of RA/CRS on VT burden, ICD interventions, and quality of life as we believe those to be the main clinical endpoints for future CRS trials to come. However, there are lots of further unknowns covering all aspects of this new treatment modality. One critical aspect is to define the adequate patient population for RA/CRS. We believe that patients suffering from severe heart failure and refractory ventricular tachycardia with limited local invasive treatment options and escalated antiarrhythmic medication but still with a life expectancy of more than 6 months will benefit most from this treatment. Currently, little is known about possible long-term sequelae of RA/CRS in patients with a medium-term and long-life expectancy and a more favorable prognosis. Most patients treated with RA/CRS so far had ischemic cardiomyopathy as the underlying structural heart disease, but also patients with non-ischemic cardiomyopathy have been treated [22].

For planning and delivery of cardiac SRS, the question of the ideal target volume to be treated and the technique used for identification of the target area has not yet been answered. Data from a randomized controlled trial of catheter ablation in patients with VT demonstrated that ablation of the whole arrhythmogenic substrate may be superior to clinical VT ablation [75]. In the early case reports and in some cases series, only a limited target area, often those areas identified by invasive electrophysiological mapping that were not accessible or unsuccessfully covered by catheter ablation, was treated [19, 25, 26, 31]. However, in the phase I/II ENCORE trial [22], external electrocardiographic mapping of the induced VT was performed and the VT exit site as well as the myocardial scar as defined by available imaging were included into the target volume [22]. Interestingly, a secondary analysis for the ENCORE trial recently found a decrease in target volume size over time/team learning curve and correlation between larger target volume and shorter overall survival [23].

Presumably, harmonization of RA/CRS can only be achieved with additional clinical data, benchmark studies, development of a method to reliably transfer electrocardiographic data to the radiation treatment planning systems, and, finally, by choosing the appropriate planning volume margins depending on the motion management strategy of each center. This is part of the quality assurance program for the RAVENTA trial and will be published subsequently. Other indications for RA/CRS may include rescue procedures for patients with electrical storm [27, 30] and potentially adjuvant procedures after catheter ablation where recurrence is likely [1–5]. If RA/CRS may play a role in the treatment of other cardiac arrhythmia is unknown at this stage, a better understanding of dose-volume and dose-rate effects in the heart for various underlying diseases and targets is needed to optimize the actual target and the necessary radiation dose in the future. Still, the cell biological mechanisms and the temporal process of the effects of RA/CRS are poorly understood. For a single fraction dose of 25 Gy, the phenomenon of marked clinical effects in terms of reduction of VT burden or ICD interventions without the clear formation of a scar has been reported from preclinical data [34, 36, 38, 39] and even from patients treatments [24, 31].

In summary, while additional preclinical studies and systematic analytical approaches are needed to determine the effects of RA/CRS on a cellular level, clinical studies are needed to answer the questions of safety, feasibility, and in the long-run efficacy while trying to fill the current void of the many open questions regarding patient selection and target volume definition and optimal treatment delivery which we face. The RAVENTA trial is thought to answer the first question in a multi-center multi-platform setting to gather data for subsequent clinical trials ultimately designed to optimize patient selection and treatment technique for RA/CRS.
